# Estimation of the Respiratory Rate from Localised ECG at Different Auscultation Sites

**DOI:** 10.3390/s21010078

**Published:** 2020-12-25

**Authors:** Xinqi Bao, Aimé Kingwengwe Abdala, Ernest Nlandu Kamavuako

**Affiliations:** 1 Department of Engineering, King’s College London, London WC2R 2LS, UK; Xinqi.bao@kcl.ac.uk; 2 Faculté de Médecine, Université de Kindu, Kindu, Democratic Republic of the Congo; a.abdala@univ-kindu.ac.cd

**Keywords:** respiratory rate (RR), Electrocardiogram (ECG), ECG derived respiration (EDR), auscultation sites

## Abstract

The respiratory rate (RR) is a vital physiological parameter in prediagnosis and daily monitoring. It can be obtained indirectly from Electrocardiogram (ECG) signals using ECG-derived respiration (EDR) techniques. As part of the study in designing an early cardiac screening system, this work aimed to study whether the accuracy of ECG derived RR depends on the auscultation sites. Experiments were conducted on 12 healthy subjects to obtain simultaneous ECG (at auscultation sites and Lead I as reference) and respiration signals from a microphone close to the nostril. Four EDR algorithms were tested on the data to estimate RR in both the time and frequency domain. Results reveal that: (1) The location of the ECG electrodes between auscultation sites does not impact the estimation of RR, (2) baseline wander and amplitude modulation algorithms outperformed the frequency modulation and band-pass filter algorithms, (3) using frequency domain features to estimate RR can provide more accurate RR except when using the band-pass filter algorithm. These results pave the way for ECG-based RR estimation in miniaturised integrated cardiac screening device.

## 1. Introduction

Respiratory rate (RR) is the physiological indicator of breaths per minute, which is commonly used as an early warning sign in disease detection. The normal RR of a healthy adult at rest is between 12–16 bpm [1]. Compared with adults, children’s RR is higher. For an infant, it ranges from 30–60 bpm, and with growth, the RR will gradually reach the adult level [2]. The resting RR of older people may slightly increase. For the healthy independent seniors, it is 12–20 bpm, and those who need long-term care will reach 16–25 bpm [3]. Generally, a resting RR outside of these ranges may indicate a potential disease. An increased RR (tachypnea) may suggest fever, dehydration, asthma, chronic obstructive pulmonary disease, heart disease, etc. [4]. A low RR (bradypnea) may reveal the use of narcotics, alcohol intake, abnormal body metabolism, sleep apnoea, etc. In critical care (or intensive care, ICU), RR is also a vital parameter in the monitoring of respiratory failure. It could be measured by the gas exchange using a ventilator, capnography monitors, or spirometry devices, and chest electrical activities using electrical impedance tomography (EIT), inductance plethysmography, or impedance pneumography [5,6].

The current measurement of RR outside of the critical care still relies on manually counting the chest undulations in one minute by the medical staff [7]. Although this practice is easy to conduct without using extra medical devices, it has some drawbacks such as low accuracy. Subject’s awareness, poor visibility of a breath, and other interruptions will greatly affect the measurement. Besides, in practice, the manual counting is not completed in one full minute by the medical staff due to the heavy workloads. They usually multiply the 30 s or 15 s measurement by 2 or 4 to assess the RR, which will lead to further inaccuracies [8]; (2) it is labour-consuming, as the medical staff can only conduct the measurement on one patient at one time; (3) the measurement is not continuous. As an early sign of physical deterioration, real-time and continuous monitoring can help alert the staff to emergencies, such as heart failure, shock, diabetic coma, etc. However, the intermittent measurement cannot provide such information timely, so the RR is always underutilized. External devices to automate the RR measurement can remedy the deficiencies associated with manual counting to a certain extent. Despite this, there are still respective limitations to each method. For the gas exchange-based techniques, they are accurate methods to reflect the respiratory condition, but have no portability, which requires the patients breathing in the external tube of the devices. So, these techniques are generally only available in critical care [9]. The bioimpedance-based techniques such as impedance pneumography can measure the electrical activities on the chest during inhalation and exhalation. However, it requires the patients to wear a tight chest strap, which may cause discomfort [10]. Additionally, patient movement, bad contact, and obstruction of breath will cause inaccurate measurements. Acoustic sensors are also used in the measurement of RR, however, their performance will be affected by the environmental noise and skin friction [11]. Therefore, wearable devices for automatic RR measurement are in great need to effectively monitor the breath in real-time and detect the first sign of physical deterioration promptly.

**Figure 1:**
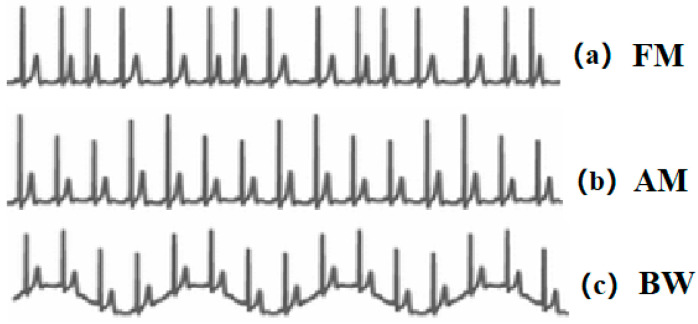
(**a**) Respiratory Sinus Arrhythmia (RSA) induced frequency modulation (FM). (**b**) Electrical axis rotation caused amplitude modulation (AM). (**c**) Baseline wander (BW) caused by chest movement.

Extracting respiratory signals from the Electrocardiogram (ECG) signals is a potential surrogate measurement of RR. In recent years, ECG devices are becoming miniaturised, and sensors have been integrated with sport bands, smartwatches, and other portable monitors. This provides the feasibility and potentiality to design wearable ECG-based RR measurement devices. The first study on respiration-induced ECG variation was proposed by Einthoven et al. [12]. Flaherty and Riekkinen further analysed the respiration influence on children and cardiac patients by isopotential surface-mapping and vectorcardiography (VCG) [13,14]. Nowadays, it is well known that respiration-induced ECG variations are caused by (1) Respiratory Sinus Arrhythmia (RSA) that refers to the cyclic variation that the heart rate accelerates during inhalation and decelerates during exhalation [15]. It can be reflected in the ECG signals as the frequency modulation (FM) of the R-R interval between the R peaks as shown in Figure 1a. (2) Respiration-induced electrical axis rotation. During the inspiration, the filling of the lungs stretches the heart apex towards the abdomen, and in expiration, the emptying of the lungs compresses the heart towards the breast. Due to the displacement of the heart, the electric cardiac vector will change during respiration [16]. In the ECG signal, this process can be indicated as amplitude modulation (AM) of the R peaks as shown in Figure 1b. (3) Baseline Wander (BW) is the artefact caused by body movement, including breathing. The expansion and contraction of the thoracic cavity due to respiration will cause a slow and undulating baseline in the ECG signals as depicted in Figure 1c [17].

Several techniques to extract respiratory signals from the ECG, the so-called ECG-derived respiration (EDR), have been proposed according to the respiration-induced ECG variation mentioned above. Some techniques are based on multi-leads ECG signals [16,17,18,19,20], while others attempt to extract respiratory information from one-lead ECG [21,22,23,24,25,26], as well as direct band-pass filtering (BP) of the ECG within the respiratory frequency band [17,26,27]. For the multi-leads EDR techniques, they mainly use the rotation angles of VCG from multiple ECG leads, while the one-lead EDR methods focus on the features related to the QRS complex, such as amplitude, interval, area, slopes, etc. There is no consensus on which is better in the performance; however, for a wearable device, one-lead ECG has the advantage in the system complexity and size. As part of our long-term project to design an integrated device for early cardiac screening, the final aim is to propose a small integrated device (around 8 cm^2^) that can provide multiple physiological parameters including heart sound, ECG, and RR. The device will measure the ECG locally with heart sound rather than at different body parts. In our previous study, we did experiments to analyse the time property between ECG and heart sound when the ECG is captured at different auscultation sites [28]. Additionally, it indicates that the location of the ECG will cause the morphological variation of its signal, which may affect the obtainment of the EDR signal, therefore an important motivation of this study is to further analyse if these ECG variations will affect the performance of the EDR algorithms under this condition.

The aim of this study is threefold: (1) To investigate if the location of the electrodes at auscultation sites will affect the EDR algorithm accuracy; (2) to compare the performance of one-lead EDR algorithms based on the mentioned respiration-induced ECG variation; (3) to compare time-domain and frequency-domain features for RR estimation. All the findings will contribute to providing more accurate RRs for the integrated cardiac screening device.

## 2. Methodology

### 2.1. Subjects

The experiments were conducted on 12 healthy human subjects (8 male/4 female, age range 21–29 years, mean 25.9 years) with no history of heart diseases or respiratory issues. The procedures were approved by the King’s College Research Ethics Committee (Approval No.: LRS-18/19-10673). Subjects gave written informed consent before the experimental procedures.

### 2.2. Experimental Setup

**Figure 2:**
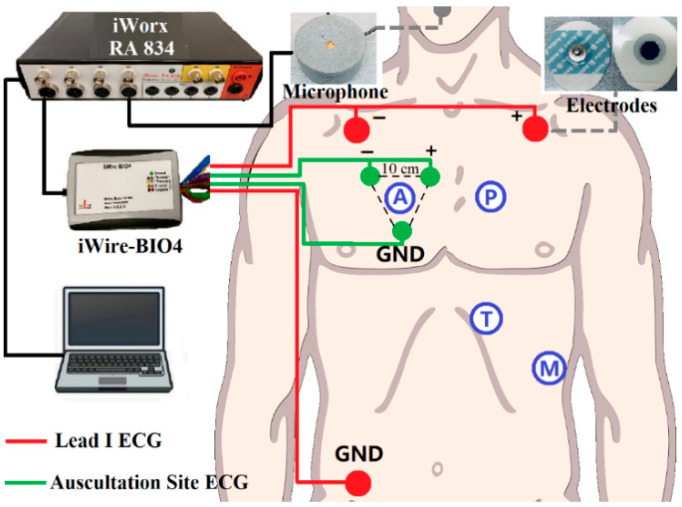
Block diagram of the recording setup: Red dots are Lead I Electrocardiogram (ECG) as a reference, green dots are auscultation site ECG. The grey dot is the microphone for respiration recording. iWire BIO4 is for ECG recording. All the data is transferred to the computer for processing through iWorx RA 834.

The standard Lead I ECG (as reference ECG), auscultation site ECG (captured at auscultation site A, P, T, M with 10 cm inter-electrode distance), and respiratory signals were recorded simultaneously during the experiment. A simple block diagram of the experimental setup is shown in Figure 2. The sensors for ECG signals were solid gel electrodes (Ambu WS, size: 36 × 40 mm, Medico Electrodes International LTD., Uttar Pradesh, India), and the respiratory signal was captured by a small microphone (developed at the Centre for Robotics Research (CORE) at Kings College London, UK) placed under the subject’s nose. The recording used the commercial acquisition system (iWorx, model RA834, iWorx Systems Inc, Dover, NH, US) and ECG devices (iWire-BIO4, iWorx Systems Inc, Dover, New Hampshire, US). The sampling frequency was 1 kHz and the analog filter for the ECG was 0.05–40 Hz [29].

During the experiment, subjects should keep supine and remain calm. Besides, subjects were required not to make sound from the larynx to ensure the sound captured was only respiration. The Lead I ECG and different auscultation site ECG signals were measured in pairs together with the respiratory signals. The duration of each recording group was three minutes, and two minutes break was given between different auscultation site trials.

### 2.3. Signal Processing

In this study, EDR signals were obtained using BW, AM, FM, and BP algorithms from the reference (Lead I) and auscultation sites ECG signals, respectively. The RRs were estimated from the EDR signals using time and frequency domain features as detailed later. The performance of the algorithms and the effect of the locations were analysed by comparing it with the measured respiratory rate. The processing was conducted in the Matlab ^®^ R2018b environment, and the statistical analysis was performed using IBM ^®^ SPSS version 26.

#### 2.3.1. Signal Filtering

The captured ECG signals and respiration sounds were filtered first to remove the unwanted artifacts and noise. For the ECG, a zero-phase 3rd-order Butterworth high-pass filter at 0.1 Hz was used to eliminate the large artifacts which were not related to respiration [30]. For the respiration sound, a 3rd-order Butterworth band-pass filtered (0.1–0.5 Hz) was used to smooth the waveform.

#### 2.3.2. EDR Signals Extraction

In AM, BW, and FM algorithms, *R*-peak detection was a vital step, as all the features to be captured were related to R peaks. In this study, the Pan–Tompkins algorithm was used to detect *R*-peaks in the ECG signals [31].

1. AM algorithm: The amplitude changes due to the respiration in the ECG signals was obtained by connecting the captured *R*-peaks.
2. BW algorithm: Based on the *R*-peaks, Q points were found using the gradient descent method. Then, the baseline wander could be generated by connecting the middle points between *R*-peaks and Q points [32].
3. FM algorithm: The intervals between the R peaks were calculated. The resulting signal was the frequency modulation caused by respiratory sinus arrhythmia.

Afterward, all the signals generated by the algorithms above were interpolated to the same sample size of its raw ECG signals to increase the resolution.

4. BP algorithm: A band-pass filter (0.1–0.5 Hz) was used to capture the EDR signals. Although the normal RR for a healthy adult ranges between 0.2–0.35 Hz at rest, in our processing, we appropriately expanded the range to enable it to respond to special situations, such as the subjects’ occasional deep or rapid breaths. Besides, a wider band can help to further analyse the frequency components when there are no dominant peaks.

**Figure 3:**
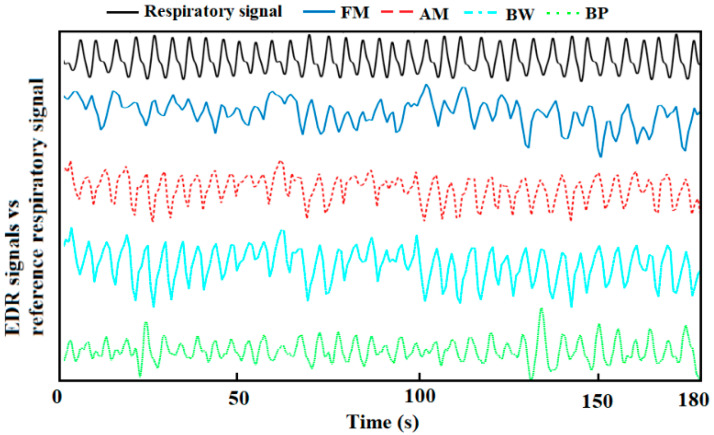
A representative derived respiration signals from auscultation site ECG and reference respiration signal.

Representative derived respiration signals by the methods above are shown in Figure 3.

#### 2.3.3. Respiratory Rate Estimation

The reference RRs were obtained from the filtered respiration sound recorded using a nostril microphone. It was manually counted in the waveform to ensure accuracy. The estimated RRs from EDR signals were calculated by automatically counting in the time domain and using the median frequency (between 0.1–0.5 Hz), respectively. For the counting method, a moving average filter (window length: 50 ms) was used first to smooth the EDR signals and eliminate sub-peaks. Then, peak detection with the threshold of the signal mean value provided the estimated RR. The median frequency was chosen according to our previous study, which was proven to be the best feature in the frequency domain to estimate RR from EDR signals [27].

### 2.4. Statistical Analysis

The mean absolute errors (MAE) between the EDR-based estimated RR and reference RRs was used as the performance measure provided as mean ± standard error (SE). A three-way repeated-measures analysis of variance (ANOVA) was used to compare MAE. Factors were the features (counting and median frequency), EDR algorithms (AM, BW, FM, BP), and ECG locations (A, P, T, M, Lead I). A *P*-value of less than 0.05 was considered significant. Data were log-transformed to obey normality and variance homogeneity was satisfied.

## 3. Results

### 3.1. ECG Morphological Variation among the Auscultation Sites

**Figure 4:**
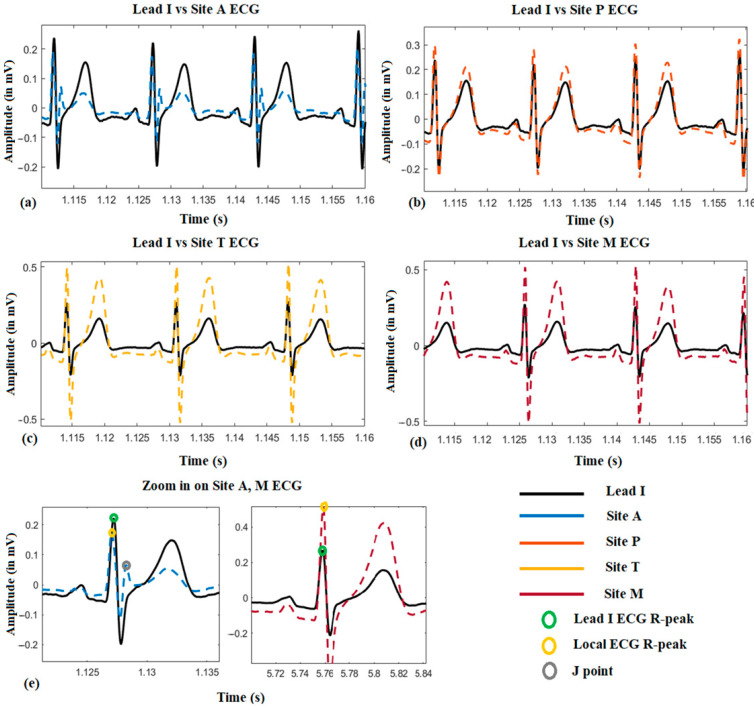
The local ECG morphological variation compared with reference Lead I ECG.

Figure 4 shows a representative local ECG morphological variation compared with the lead I ECG from one subject. From (a) to (d), it can be seen that the amplitude of the *R*-peak, *s*-wave, and *T*-wave become larger from auscultation site A to M. Besides, it is also found that the *R*-peak of the site A ECG is normally on the left-hand side of it on Lead I ECG, which means the *R*-peak is advanced (approximately 10 ms by average). However, it will shift to the right-hand side when measured at site M, which means its onset is delayed (approximately 15 ms by average). Another phenomenon could also be observed that in the site A ECG, there is a J-point elevation shown as grey dots in (e). This happened on five subjects, and in 3 of them, the J point is even higher than the *R*-peak.

### 3.2. Location Effect on EDR among the Auscultation Sites

**Figure 5:**
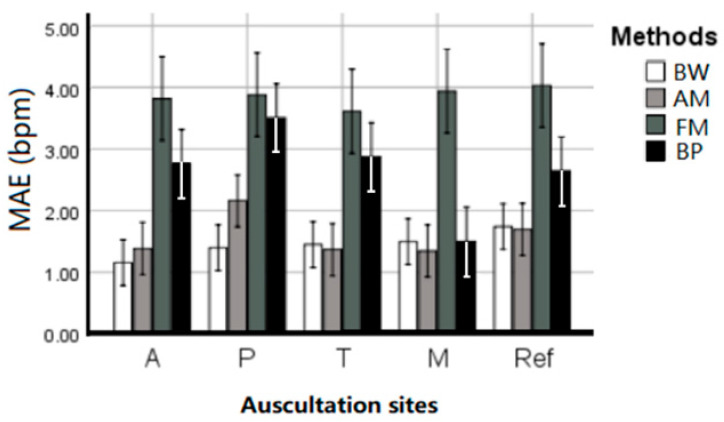
The performance (MAE ± SE bpm) of the EDR algorithms on different auscultation sites and Lead 1 ECG signals.

Table 1 summarized the EDR MAE of each subject averaged across estimation techniques and given per auscultation site. ANOVA results indicate that there is no statistical difference between the five sites (*p* = 0.746), and there was no interaction between EDR algorithms and sites (*p* = 0.516). All four EDR algorithms have quite close MAE between each auscultation sites, including average MAE at A: 1.656 ± 0.351, P: 2.297 ± 0.476, T: 1.733 ± 0.461, M: 1.467 ± 0.326, and reference ECG (Lead I): 1.834 ± 0.378 bpm. This indicates that RR can be harvested using ECG anywhere on the chest with negligible location effect. Figure 5 further visualized the location effect with different algorithms.

### 3.3. The Performance of the EDR Algorithms

After statistical analysis of the MAE on each subject with different EDR methods shown in Table 2, there was a significant difference between the four EDR algorithms (*p* < 0.001). The BW algorithm performed with MAE = 1.446 ± 0.181 bpm, closely followed by the AM algorithm with 1.589 ± 0.1966 bpm. Post hoc analysis revealed no statistical difference between BW and AM (*p* = 0.31), however, they were both significantly better (*p* < 0.05) than BP (MAE of 2.656 ± 0.258) and FM (MAE of 3.855 ± 0.329 bpm).

### 3.4. Time vs. Frequency Domain

**Figure 6:**
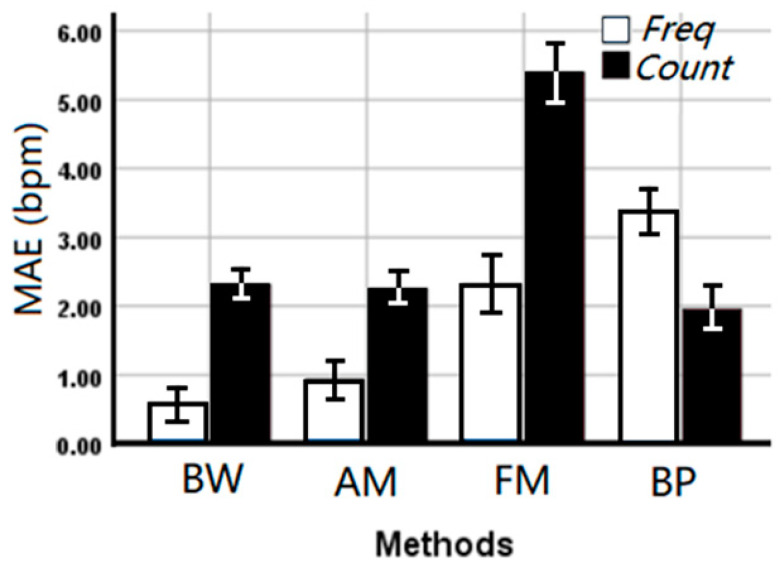
The mean absolute error (MAE ± SE bpm) of the EDR rates between EDR algorithms and estimation methods. Freq: Median frequency in the frequency domain, Count: Automatic counting in the time domain.

Deriving respiration rate using the median frequency (overall MAE 1.80 ± 0.223 bpm) outperformed the counting method (overall MAE 2.98 ± 0.312 bpm) in the time domain (*p* < 0.001) suggesting stability of the frequency domain, although a significant interaction (*p* < 0.001) with the applied method was observed. From Figure 6, it can be seen that the median frequency can provide a more accurate estimated RR on BW, AM, and FM algorithms. However, counting in the time domain is more accurate for the BP algorithm.

## 4. Discussion

This study aimed at analysing the performance of one-lead EDR algorithms in auscultation site ECG signals and EDR rate estimation in both time and frequency domain. The results show:

Firstly, it is found that the location effect on the obtainment of EDR between auscultation site and Lead I ECG signals is negligible in our experimental data. The result revealed that the ECG morphological variation between auscultation sites happened on the onset and amplitude of the ECG components including the *R*-peak delayed from site A to M, and the amplitude increase of *R*-peak, *s*-wave, and *T*-wave. These won’t directly affect the EDR signals extraction, but it is worth noticing in cardiac researches. It is still unclear on the occurrence of J-point elevation or RSR’ (An ECG finding in which there are two R waves) in five subjects’ site A ECG signals. Normally they are pathological, but the subjects were confirmed healthy with no heart conditions, and this can be normal for the age group. The high J-point or double *R*-peaks may interfere with *R*-peak detection when the fake *R*-peak is higher than the true one. In our study, the performance of the four chosen EDR techniques was not affected, however, it may have an impact on the QRS area or slope based EDR methods. In the study of Sakai, it indicated that the location of the electrodes affected the quality of EDR signals and the more accurate RR estimation was obtained when the electrodes were attached near the heart [22]. The best placement was a negative electrode at the bucket-handle and a positive electrode at pump-handle movements of the ribs. However, in our experiment, electrodes were placed at auscultation sites on the upper chest, which were already close to the heart. Besides, as we want to design a miniaturised device, the inter-electrode distance was fixed and short (10 cm). Therefore, from the physiological mechanism, the locations in our study barely have an effect on the respiratory sinus arrhythmia, and the effect on the respiration-induced electrical axis rotation and chest undulation-induced baseline wander are minimal. This result verifies that the location effect on RR estimation can be ignored in designing an integrated cardiac screening device.

Secondly, the BW and AM algorithms outperformed FM and BP algorithms. Although BW has a slightly smaller MAE (1.446 ± 0.181 bpm) than AM (1.589 ± 0.1966 bpm), the difference (*p* = 0.315) is not statistically significant in our experiment data, which cannot confirm that the performance of BW is better than AM so far. This is in contrast with some previous work. In Charlton’s study, it was shown that the BW performed better than AM without statistical analysis [7]. The performance of FM and BP methods are in line with previously reported MAE using the PhysioNet’s MIMIC-II database, while the results of AM obtained in this study are similar to the MAE reported by Widjaja et al. [26] using their experimental data. It seems like the experimental setting for the database had a significant impact on the performance of EDR algorithms. Because our experiments were conducted under ideal conditions where the subjects were required to lie down calmly without any movement, the MAE was much lower than studies that have made use of the database.

For respiratory sinus arrhythmia induced FM, the magnitude of the oscillation varies from individual to individual, so that the obtained EDR signal is not that conspicuous sometimes [33]. For example, the FM waveform of 0–50 s is shown in Figure 3, the EDR signal in that period is messy, thus it will dramatically affect the peak detection in the time domain, causing inaccurate RR estimation. That should be the reason for FM’s poor performance. For the BP algorithm, the choice of the frequency band is the current limitation. Though the frequency band (0.1–0.5 Hz) used in this study is appropriately extended, it is still not enough to capture RR from young children and stress tests. Besides, the use of a simple band-pass filter cannot remove unwanted interferences completely. The low-frequency component between 0.1–0.2 Hz, which is related to the baroreceptor reflex (blood pressure is regulated by the baroreceptors through the autonomic nervous system) and the high-frequency harmonic between 0.4–0.5 Hz will interfere with the RR estimation in the frequency domain [34]. Therefore, an adaptive frequency band is essential to improve the performance of using a band-pass filter.

Thirdly, the RR estimation in the frequency domain is found to be better than the time domain for BW, AM, and FM [35]. This result is the opposite of Charlton’s result, which said Fourier analysis was inferior to breath detection in the time domain [7]. As discussed above, there are conditions where the EDR is not conspicuous enough, thus in the time domain, it is hard to detect the corresponding respiration related peaks, while still possible to capture it based on the power spectral density function. Besides, at the beginning and end of the EDR signals, there may be incomplete breathing, this will lead to the error for counting in the time domain. As there are not many breaths per minute, these errors are considerable for the RR estimation. Using frequency features will reduce this error moderately. However, it is also noticed that the performance of frequency estimation for the band-pass filter is worse than counting in the time domain as there are mentioned lower-frequency and higher-frequency components in the spectrum which weaken the domination of the respiratory band. Therefore, further analysis of frequency components is needed to improve accuracy when using the BP algorithm.

In this study, our research focused on the EDR of healthy adults at rest, and the experimental conditions were ideal that the subjects kept supine and breathed evenly without any movement. However, there are conditions of practical application that need to be considered including EDR performance on irregular respiration, such as deep breath or an increased respiration rate. Improvement still can be done to improve the RR estimation accuracy. The current validation study has compared four algorithms of the existing algorithms. More algorithms and fusion methods could be tested to improve the accuracy for clinical use. Future studies will include RR estimation throughout monitoring via a Holter-like monitor.

## 5. Conclusions

This study analysed the location effect on EDR algorithms’ performance between auscultation sites and compared four EDR algorithms to estimate RRs in the time and frequency domain. The results showed that, firstly, the location of the ECG electrodes between auscultation sites barely affects the estimation of RR. Secondly, the BW and AM algorithms outperformed than FM and BP algorithms in generating the approximation of the respiratory signal. Thirdly, RR estimation in the frequency domain is more reliable except on BP algorithms. All the findings will contribute to building chest-based multiple physiological parameter monitors and providing more accurate RR estimation using EDR algorithms.

## Figures and Tables

**Table 1:** The ECG-derived respiration (EDR) mean absolute errors (MAE) of each subject for each auscultation site and Lead 1 ECG signals, averaged across estimation techniques.

	A	P	T	M	Lead I
Subject1	0.16	0.06	0.14	0.12	0.08
Subject2	2.27	3.74	1.77	0.86	2.00
Subject3	1.88	1.37	3.17	2.15	2.60
Subject4	0.50	1.69	0.54	0.82	0.83
Subject5	1.27	1.10	0.28	0.14	0.47
Subject6	5.79	4.61	6.91	3.23	5.45
Subject7	1.60	2.60	0.38	2.60	2.24
Subject8	2.54	5.41	3.16	3.47	2.24
Subject9	0.73	0.20	0.67	0.53	1.16
Subject10	0.36	1.62	1.69	1.25	1.11
Subject11	0.85	2.10	1.03	1.46	1.45
Subject12	1.93	3.06	1.07	0.99	2.39
Mean	1.66	2.30	1.73	1.47	1.83

**Table 2:** The EDR MAE of each subject on different EDR methods, averaged across auscultation sites.

	BW	AM	FM	BP
Subject1	1.61	1.92	5.86	1.93
Subject2	1.65	2.88	3.26	2.45
Subject3	1.33	1.82	2.04	0.47
Subject4	2.19	1.99	4.06	2.19
Subject5	0.91	0.92	1.54	2.93
Subject6	0.49	0.41	3.55	3.67
Subject7	0.34	0.66	0.73	0.86
Subject8	0.76	0.75	3.23	1.19
Subject9	3.98	3.54	11.93	6.75
Subject10	0.45	0.38	2.08	0.38
Subject11	1.81	2.02	3.36	3.59
Subject12	1.84	1.78	4.61	5.89
Mean	1.45	1.59	3.85	2.69

## Data Availability

Data sharing not applicable.
